# An mHealth app technology to strengthen adverse event management of multi‐drug‐resistant tuberculosis in Vietnam: Protocol for a process evaluation of the V‐SMART trial

**DOI:** 10.1111/tmi.14091

**Published:** 2025-02-16

**Authors:** Binh Hoa Nguyen, Tho T. H. Dang, Duy Trinh Hoang, Thu Thuong Do, Luong Van Dinh, Viet Nhung Nguyen, Dinh Hoa Vu, Dorothy Drabarek, Tram N. B. Nguyen, Dang Vu, Thu Anh Nguyen, Guy B. Marks, Joel Negin, Greg J. Fox, Sarah Bernays, H. Manisha Yapa

**Affiliations:** ^1^ The National Lung Hospital Hanoi Vietnam; ^2^ Hanoi Medical University Hanoi Vietnam; ^3^ Woolcock Institute of Medical Research New South Wales Australia; ^4^ Faculty of Medicine and Health The University of Sydney New South Wales Australia; ^5^ National Drug Information and Adverse Drug Reaction Monitoring Centre Hanoi Vietnam; ^6^ School of Clinical Medicine University of New South Wales Sydney New South Wales Australia; ^7^ London School of Hygiene & Tropical Medicine University of London UK; ^8^ Sydney Infectious Diseases Institute The University of Sydney Sydney New South Wales Australia

**Keywords:** tuberculosis, adverse event management, digital health, mHealth, multi‐drug‐resistant, *Mycobacterium tuberculosis*, process evaluation, resource‐limited

## Abstract

**Background:**

Drug‐related adverse events cause poorer treatment outcomes amongst people with multi‐drug‐resistant tuberculosis, exacerbating a major global public health problem. The *Harnessing new mHealth technologies to Strengthen the Management of Multi‐Drug‐Resistant Tuberculosis in Vietnam* (V‐SMART) trial tests whether a mobile health (mHealth) application (app) can optimise management of drug‐related adverse events, within routine health services in Vietnam. Implementation of digital health within routine services is complex and driven by behaviour change as well as a range of health system factors. Understanding implementation is key to informing the evidence base for digital health prior to scale up, despite its potential appeal.

**Methods:**

Through a process evaluation of the V‐SMART trial, we aim to (i) understand the multi‐drug‐resistant tuberculosis service delivery context and how trial procedures are implemented within services; (ii) describe ‘dose’ and ‘reach’ of the app; and (iii) understand health worker and patient perspectives of app implementation and identify areas for improvement.

To achieve this, we will (i) conduct process maps (patient flow maps) to describe implementation of the mHealth intervention within routine multi‐drug‐resistant tuberculosis health services including adverse event management pathways at different levels of the health system; (ii) measure app usage by all participating health workers and people with multi‐drug‐resistant tuberculosis over time; and (iii) conduct a total of up to 45 semi‐structured interviews in seven provinces, with people with multi‐drug‐resistant tuberculosis, health workers, and policymakers, to identify determinants of app uptake and suggestions for future person‐centred app design. Interview topic guides are informed by the Theoretical Framework for Acceptability, Normalisation Process Theory, and the Tailored Implementation of Chronic Diseases framework respectively.

**Discussion:**

The process evaluation will strongly complement the parent trial impact evaluation, and the economic evaluation. Moreover, it will inform future tailored approaches to scaling up digital health as part of broader health system strengthening initiatives.

## BACKGROUND

Multi‐drug‐resistant, including rifampicin‐resistant tuberculosis (RR/MDR‐TB) poses a major global public health threat, affecting almost half a million people annually [1]. The second‐line antibiotic therapies required to treat RR/MDR‐TB are often poorly tolerated, and toxicity remains a major barrier to successful outcomes [2, 3]. Some drugs previously used as part of WHO‐recommended combination therapies to treat drug‐resistant—including extensively drug‐resistant‐TB—such as para‐aminosalicylic acid, prothionamide and injectable agents (including amikacin), frequently cause considerable short and long‐term morbidity [4], and require prolonged courses of at least 18 months. Over half of all people treated for RR/MDR‐TB with these previous regimens experienced significant side effects including nausea, abdominal pain, hearing loss, kidney impairment and psychiatric disorders [3, 5, 6].

Newer oral regimens—containing bedaquiline, pretomanid and linezolid—are better tolerated and administered over shorter (6–9 months) durations for successful RR/MDR pulmonary TB treatment [4, 7]. Vietnam commenced rollout of bedaquiline‐containing regimens for people with RR/MDR‐TB in 2021. However, even these newer regimens can cause serious adverse events, such bone marrow suppression and peripheral neuropathy [8, 9]. Thus, all people treated for RR/MDR‐TB require careful pharmacovigilance during treatment, and early intervention to minimise attrition from care, treatment interruption and onward transmission of *Mycobacterium tuberculosis*.

People with RR/MDR‐TB may experience delays accessing health services to address their adverse events, particularly if they must travel long distances to care or require referral to more specialised services at higher levels of the health system. In recognition of the need to improve adverse event management in a timely manner—and improve RR/MDR‐TB treatment success—the V‐SMART trial was established to evaluate the effectiveness of a mobile health (mHealth) application (app) upon treatment outcomes in Vietnam [10]. Participant recruitment to this open‐label trial commenced in October 2020, after the onset of the COVID‐19 pandemic in Vietnam.

During initial implementation of the intervention, research staff observed barriers to the uptake and usage of the smartphone app. Participants reported using alternative communication modalities and challenges with the app user interface. Health worker compliance with the intervention varied considerably between settings. Qualitative research conducted in four provinces in the early phase of the trial, demonstrated that app use depended upon resources, technical abilities of patients and health workers, and the perceived relevance to health workers' day‐to‐day clinical practice (manuscript in preparation). Process mapping was also implemented alongside the early‐phase qualitative research to better understand app implementation within health services. Findings of the qualitative research and process mapping informed a series of modifications to the app including streamlined messaging and frequency of login reminders, and strengthening ease of app navigation and information in frequently asked questions.

Despite these modifications to the app, we continue to observe low app use amongst trial participants and health workers. It is therefore crucial to understand the extent to which implementation challenges may influence engagement with the app, and how best to implement such technologies within routine health services in future iterations.

We will therefore undertake a comprehensive process evaluation to understand implementation of the V‐SMART ‘Bac Sy Minh’ app within routine RR/MDR‐TB services in seven provinces in Vietnam. Importantly, we will include process pathways of adverse event management within the health system, as this was the main target of the smartphone app. Findings will complement the parallel economic evaluation of the V‐SMART trial [11] to inform future design and sustainability of the technology, using a person‐centred approach.

## METHODS

### Overview of study setting and V‐SMART trial

TB services in Vietnam are overseen by the National TB Program (NTP), whilst RR/MDR‐TB services fall under the auspices of the national Programmatic Management of Drug‐resistant TB (PMDT). Public sector TB services are provided at primary care, district, provincial and national level. Resources (including laboratory) and clinical expertise for diagnosing, initiating RR/MDR‐TB treatment and serious adverse event management, are available at provincial or national level facilities; conversely, diagnosis and treatment initiation for drug‐susceptible TB can occur at district level facilities. Routine treatment and adherence monitoring occur at lower levels of the health system (district and commune health facilities).

The V‐SMART randomised controlled trial and intervention have been described in detail elsewhere [10]. Briefly, the smartphone app facilitates communication between health workers and people on RR/MDR‐TB treatment for timely management of adverse events—this occurs by allowing patients to report any symptoms of concern to their allocated health worker, directly via a messaging interface, or indirectly via a symptom reporting algorithm. The app alerts health workers to serious symptoms logged via the app, prompting them to contact the patient. The app also enables people with RR/MDR‐TB to record their medication adherence and alerts them to login to the app daily for 6 days per week, whilst on treatment. The trial database logs app usage patterns as part of its design.

This open label trial is being conducted at outpatient clinics in seven provinces (Hanoi, Ho Chi Minh City, Thanh Hoa, Da Nang, An Giang, Can Tho and Tien Giang) in Vietnam, at health facilities under the auspices of the government‐run PMDT [10]. Participating health facilities in V‐SMART include referral hospitals (provincial or national) and district‐level hospitals, but not primary care or community services (Table 1). Trial participants are followed up for up to 24 months after randomisation [10]. Health workers deliver the intervention embedded within routine RR/MDR‐TB services, including recruitment and interaction via the app.

**TABLE 1:** Eligibility criteria for recruitment to process mapping and interviews.

Inclusion criteria	Exclusion criteria
Individual semi‐structured interviews	
People with RR/MDR‐TB (intervention adherent or non‐adherent)	
Aged ≥18 years; AND	Aged <18 years; OR
Enrolled in intervention arm of V‐SMART Trial or declined to participate; AND	Unable to autonomously consent to participate
Up to 12 weeks have passed since last logged into mHealth app or the patient did not start using the app at all; OR	
Up to 24 weeks have passed since last logged into the mHealth app for those who are underrepresented when the above criterion is applied; OR	
Declined to participate in the trial but were invited to enrol in the trial ≤24 weeks prior to the date of proposed interview	
Health workers (doctors or nurses)	
Aged ≥18 years; AND	Aged <18 years; OR
Working at a PMDT clinic participating in the V‐SMART trial	Unable to autonomously consent to participate
Policymakers (National TB Program or Ministry of Health)	
Aged ≥18 years; AND	Aged <18 years; OR
Involved in TB policy for at least 6 months; AND	Unable to autonomously consent to participate
Aware of V‐SMART trial and its objectives	
Process mapping	
Health workers	
Aged ≥18 years; AND	Aged <18 years; OR
Working at a PMDT clinic participating in the V‐SMART trial; AND	Unable to autonomously consent to participate
Involved in implementation of the V‐SMART trial; AND	
Provide care to people with RR/MDR‐TB (e.g., doctors, nurses, public health professionals)	

The primary endpoint is RR/MDR‐TB treatment success (a composite primary outcome of bacteriological cure or treatment completion). A total of 903 participants have been randomised and the last participant is due to complete active follow‐up in August 2025.

### Process evaluation aims and objectives

**FIGURE 1:**
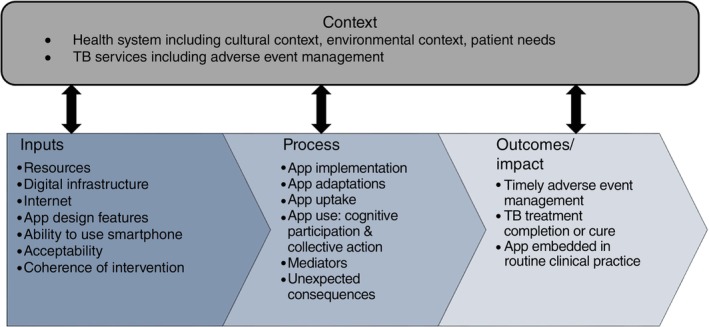
Overview of process evaluation data collection and analysis framework.

To describe implementation of the V‐SMART mHealth digital adverse event support app within routine MDR TB services. This includes describing adverse event management pathways for people on RR/MDR‐TB treatment within routine services, identifying determinants of app uptake, and identifying areas for future person‐centred app design, whilst considering the broader health system context (Figure 1).

#### Understand RR/MDR‐TB service delivery context and how trial procedures are implemented within services: process mapping of health services

Process mapping (patient flow mapping) [12] is a structured tool used in the Langley Model for Improvement and other quality improvement initiatives [13]—it enables defining essential resources and pathways involved with service delivery. We will map service delivery pathways for RR/MDR‐TB diagnosis and treatment, and adverse event management and reporting. We will then qualitatively compare differences in RR/MDR‐TB adverse event management between those with RR/MDR‐TB versus those with drug‐susceptible TB. We will also qualitatively compare differences in these pathways across participating provinces. These mapped pathways will facilitate our understanding of how the V‐SMART app fits into routine health services and the broader health system.

#### Describe ‘dose’ and ‘reach’ of the intervention: app usage amongst health workers and people with RR/MDR‐TB over time

Drawing on app usage data from the trial database, we will describe the percentage of all participating health workers logging into the app at least once per week, and at least once per month.

Amongst all people with RR/MDR‐TB enrolled in the trial, we will describe the actual logins per week as a proportion of that expected (e.g., 6 days per week for 100% adherence to intervention). We will summarise trends in app usage over the expected course of treatment, overall and by province. To measure ‘reach’ of the intervention, we will describe the proportion of eligible individuals who were recruited to the study for randomisation.

#### Understand health worker and patient perspectives of app implementation and identify areas for improvement

Through qualitative interviews, we will identify factors influencing app delivery, whether the intervention was delivered as originally intended (‘fidelity’), acceptability to app users (health workers, patients and policymakers), design ideas for future mHealth interventions, and potential facilitators of scale‐up of future mHealth interventions within the National TB Program.

The framework depicts the anticipated pathways to impact (better health outcomes and sustainability of the intervention), and how the proposed process evaluation will capture data on elements of the pathways to impact. Figure adapted from Moore et al. [14] and includes domains from Normalisation Process Theory [15], the Tailored Implementation of Chronic Diseases checklist [16], and the Systems Thinking Framework [17].

### Process evaluation study design

This mixed‐methods process evaluation will be conducted at all seven provinces participating in the V‐SMART trial. We will conduct *process mapping*, which will involve structured interviews and clinic walk‐throughs with health workers to gain facility level understanding of patient flow from TB diagnosis through to treatment and follow‐up, and adverse event management pathways (including referral systems and health worker communication modalities with each other and with patients).

By summarising *app usage* amongst all randomised trial participants and health workers, we will obtain an overarching view of ‘reach’ and ‘dose’ of the app: this is particularly important, to identify the extent to which adherence to the app (for people with RR/MDR‐TB this is logging in 6 days per week) may or may not influence adverse event management.

We will also conduct *semi‐structured interviews* to explore individuals' perceptions and experience of engaging with the smartphone intervention. Apart from a qualitative assessment of intervention fidelity, the interviews will identify how digital health interventions may support health workers with adverse event management across different levels of the health system. These findings will help ascertain potential sustainability and suitability for scale‐up of future mHealth interventions within the National TB Program.

### Study sites and participant selection: Process mapping and semi‐structured interviews

Study sites will be selected from amongst the PMDT clinics participating in the V‐SMART trial. Participants will be selected from all seven provinces, prioritising provinces that did not participate in the early‐implementation phase qualitative research. Sites from provinces previously participating in the early‐implementation phase qualitative research will also be included, to enable a comparison of processes and progress from earlier in the trial with the more recent findings—this will also facilitate a comparison of participant perceptions after the app modifications went live.

For all interviews, health facilities will be chosen to allow for maximum participation and in‐person interviews where possible. If in‐person interviews are not feasible, the interview will be conducted online using a video link such as Zoom or Teams based on participant preference. One or two additional sites will be chosen based on purposeful participant sampling, depending on recruitment to the initial selected site(s) within a given province.

### Study population: process mapping and semi‐structured interviews

The process mapping exercise will be performed by a V‐SMART research staff member with a health worker who is familiar with the operation and structure of TB services within clinics participating in the V‐SMART trial and how TB services fit in with other services provided by the clinics.

We will conduct semi‐structured interviews with four groups of participants, representing different stakeholders and therefore a range of potential reasons for app usage patterns:

1. People with RR/MDR‐TB enrolled in the intervention arm of the trial who (a) have consistently used the app over the first 4 months or treatment, and (b) those who stopped using the app regularly.
2. People with RR/MDR‐TB who declined to participate in the trial who are receiving treatment in participating PMDT clinics.
3. Health workers participating in the trial who are (a) responsible for follow‐up of people with TB identified with adverse events via the app, and (b) were caring for people with TB using the app but have since stopped using the app.
4. Policymakers from the National TB Program or National Drug Information & Adverse Drug Reaction Centre (NDIADRC).

Eligibility criteria are detailed in Table 1.

### Sample selection

*Qualitative (process mapping and semi‐structured interviews)*: People with RR/MDR‐TB enrolled in the trial, and health workers, will be selected initially using a purposive approach for maximum variation in terms of age, gender, patterns of app use (as defined above for intervention ‘adherent’ or ‘non‐adherent’), and residing in a rural or urban area. This will be determined from data gathered from the trial database on app usage, and participant information on the trial database. Subsequent sampling will be driven by emerging theoretical concerns. People with RR/MDR‐TB who declined to participate in the trial will be sampled using a convenience approach. Selection of health workers for semi‐structured interviews and process mapping will take a purposive (based on period of app use, type of health worker (i.e., doctor, nurse) and gender) and pragmatic approach (availability, workload). Policymakers will be purposively sampled.

*Quantitative*:All (*n* = 903) people with RR/MDR‐TB recruited to the parent trial for randomisation, regardless of province or health facility.

### Recruitment methods: process mapping and semi‐structured interviews

#### Process mapping

V‐SMART study staff will contact health workers by phone or email. A participant information sheet and consent form will be given to each participant prior to the interview. Written informed consent will be obtained prior to interview. If an in‐person interview cannot be conducted, then a weblink to the participant information sheet and eConsent form will be sent to them via email or mobile phone text message (SMS).

Process mapping will be conducted with one consenting health worker from each facility at which a health worker was also recruited for a semi‐structured interview—this information will help triangulate contextual information with semi‐structured interview findings, as each facility and each province will have unique cultural and organisational ecosystems.

#### Individual semi‐structured interviews

Intervention arm participants who meet the inclusion criteria for interview will be contacted by clinic or study staff by phone notifying them of the interview and then a letter of invitation, the participant information sheet and consent form provided as for process mapping. People with RR/MDR‐TB who declined to participate in the trial will be approached about the process evaluation interviews at their usual TB care follow‐up clinic visits. Individuals interested in participating will be given a contact for V‐SMART study staff if they want additional information prior to signing the consent form. If the person with RR/MDR‐TB has not responded within 2 weeks of when study documents were sent, study staff will contact the person by phone to ask if they are interested in participating in an interview. During in‐person interviews, a participant information sheet and consent form will be provided prior to the interview in paper form.

Suitable key‐informant policymakers will be identified by V‐SMART principal investigators from the Vietnamese National TB Program. Health workers and policymakers will be contacted by study staff by phone or email, using methods as detailed for process mapping. If health workers have not responded within 2 weeks, an automated email reminder will be sent, or study staff will contact the participant by phone.

As we anticipate that some interviews will not be conducted in person, the purpose of using eConsent for recruitment is to make the process of being informed about the study and providing consent more convenient for them. During the consent process, participants will be asked to give permission for the interviews to be audio‐recorded. Should they decline, detailed notes will be taken as an alternative record.

### Sample sizes

*Qualitative*. We aim to recruit up to 32 people with RR/MDR‐TB, including if feasible, at least five people with RR/MDR‐TB who declined to participate in the trial. Additionally, we aim to recruit 10 health workers, including if feasible, at least three who are not using the app. These sample sizes were determined by the anticipated number required to reach data ‘saturation’ (when no additional information emerges to address the research question) [18, 19] whilst accounting for representation across levels of the health system involved and feasibility of data analysis. We will also interview three key‐informant policymakers (Table 2).

**TABLE 2:** Overview of districts and provinces participating in early‐phase implementation qualitative research and proposed sample sizes for current process evaluation.

Provinces participating in the V‐SMART trial	Administrative (health system) level of study site	Qualitative research early implementation phase (2022)	Process mapping early implementation phase (2022)	Process evaluation interviewees		
				Health workers	People with RR/MDR‐TB^a^	Policymakers
Hanoi	Provincial only	√	√	1	5	
Thanh Hoa	Provincial only			1	3	
Da Nang	Provincial only			1	3	
HCMC	District only (19 districts)		√	3	8	
Tien Giang	Provincial only	√		1	4	
Can Tho	Provincial only	√	√	1	4	
An Giang	Provincial + district (11 districts)	√	√	2	5	
Total				10	32	3

^a^ Same facility as health worker.

At sites where the early implementation phase qualitative sub‐study (findings summarised in Introduction and manuscript in preparation) was conducted, we will aim to interview the same health worker if possible.

*Quantitative*. All (*n* = 903) people with RR/MDR‐TB recruited to the parent trial.

### Data collection

#### Process mapping

**FIGURE 2:**
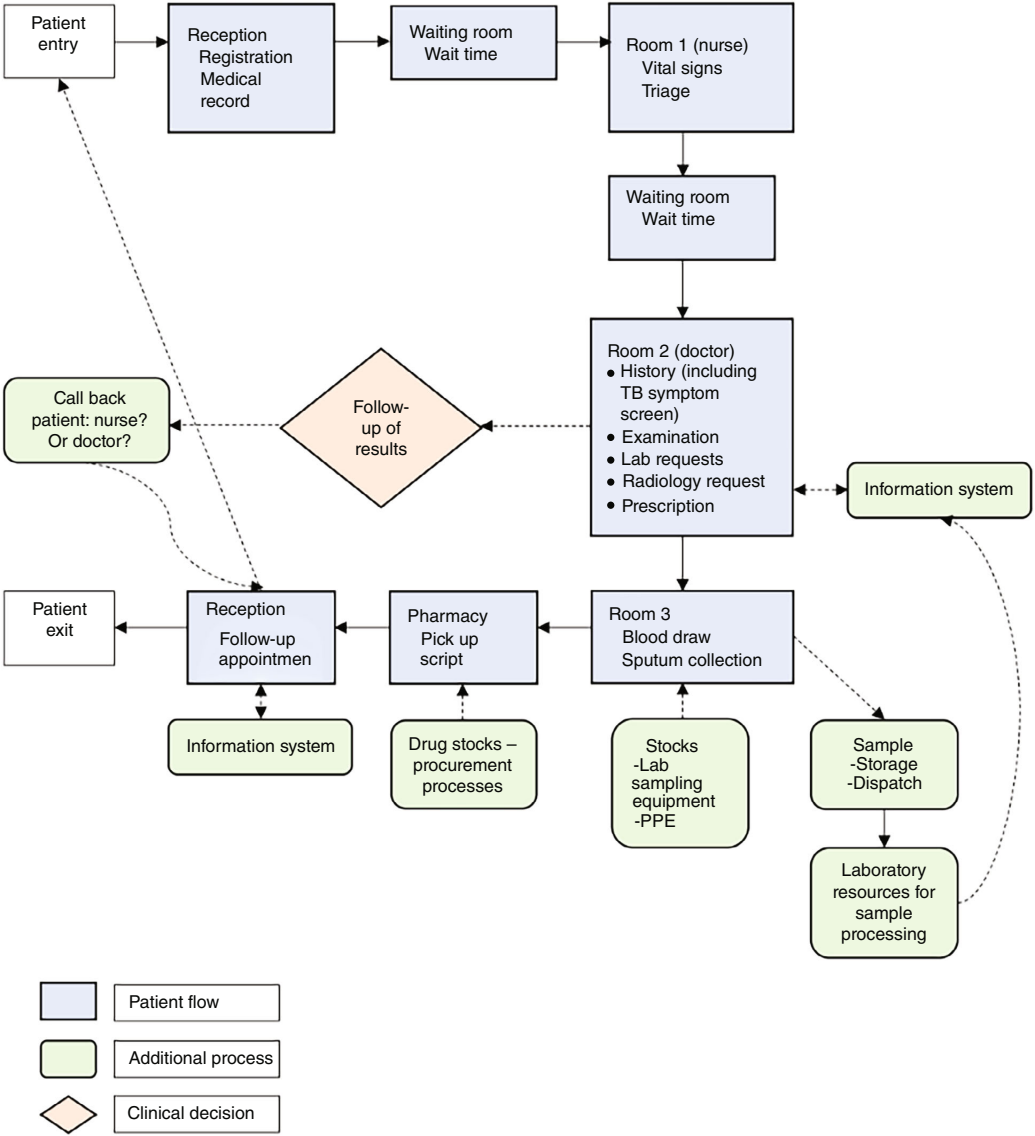
Example process map of routine TB services within a given health facility.

The process mapping interview will be conducted in Vietnamese by local trained research staff. They will use a structured questionnaire to guide the interview with a health worker and participate in a walk through the clinic guided by the health worker. The structured questionnaire (Additional File 1) responses will be audio recorded given the anticipated volume of information. The researcher will also take brief field notes during the clinic walk‐through—which will *not* be audio or video recorded to maintain patient confidentiality—to supplement and visualise information gathered during the structured questionnaire. The purpose of the clinic walk‐through is mainly to visualise clinic infrastructure and workflow such as waiting rooms and wait times between steps of a given clinic appointment (Figure 2)—clinic services or patient care will not be interrupted. The entire process mapping exercise will last approximately 60–90 min, but may be conducted over more than one appointment in order to reduce health worker burden at a given meeting.

Where a health facility previously participated in process mapping (during the early phase of study implementation), detailed questions on TB services will be avoided to minimise health worker inconvenience and duplication of information. Instead, the interviewer will briefly clarify if any major changes to TB services had occurred since the last process mapping exercise.

Each map will include a brief overview of referral systems for RR/MDR‐TB across levels of the health system (e.g., district to provincial level and vice versa) including adverse event management.

#### Individual semi‐structured interviews

Semi‐structured interviews will be conducted in local language (Vietnamese) by local trained research staff. The interviews will be informed by a flexible topic guide (Additional File 2a–c), covering keys areas of investigation. For health workers, the interview topic guide is informed by Normalisation Process Theory which facilitates understanding of how new interventions become embedded within routine clinical practice [15]. The patient interview topic guide draws on the Theoretical Framework for Acceptability [20], whilst the policymaker topic guide is informed by the Tailored Implementation of Chronic Diseases framework [16]—each framework was selected based on the research questions for the target population, such as a broader ‘health system’ overview for policymakers.

To maximise the engagement and confidence of participants in the qualitative research process, interviews will be arranged to be conducted at a time and place that is convenient for the participant. When conducted in person at health facilities, observations will gather contextual information and be documented in interview field notes. Interviews will last approximately 30–60 min. All interviews will be conducted in a language that the participant feels comfortable using.

The target sample sizes for this proposal in relation to provinces previously participating in the early‐implementation phase qualitative research and process mapping are presented in Table 2.

### Data analysis and reporting

All qualitative interviews will be audio recorded in Vietnamese and summarised in English as soon as is feasible after the interview. These will serve as detailed field notes of the interview, as well as be used to guide initial iterative data analysis conducted by the team. The audio‐recorded interviews will be transcribed verbatim in Vietnamese and translated for equivalent meaning into English, by professional translators. Transcripts will be checked by the research team for accuracy and clarity. Interview summaries will be written and shared with the investigators as soon as is feasible after each interview. This will form the basis for the team's discussions to perform the initial analyses, refine the topic guide and inform ongoing sample selection (where applicable, to further elucidate any emerging theoretical concerns). This will also serve the additional purpose of ensuring data quality.

Data analyses will be conducted by the research staff in collaboration with the investigators. A thematic coding framework will be developed, informed initially by the interview topic guide (Normalisation Process Theory, the Theoretical Framework for Acceptability, or the Tailored Implementation of Chronic Diseases checklist where applicable). We will constantly compare coding across the data analysis team to ensure consistency in coding. This will be accompanied by the iterative development of analytical memos. Qualitative data will be (descriptively) triangulated with quantitative indicators of app usage from the trial database to aid understanding of patterns of use. As this is a qualitative study with small sample sizes from each province (Table 2), we will not be able to quantitatively measure (e.g., using statistical regression) app usage patterns from the trial database against interview themes, but instead use them for descriptive purposes only.

The focus of analysis will be on providing insights to better understand barriers and facilitators of use, including a qualitative assessment of implementation fidelity (low, medium or high), and to identify whether key components of the app (qualitatively) achieved their desired utility—for example, health worker‐patient communication, better monitoring of adherence, better patient information. Moreover, we will also qualitatively consider COVID‐19 pandemic related disruptions to services, and policy changes (such as timing of rollout of new shorter‐ and better‐tolerated oral treatment regimens) when interpreting the data. These findings will aid subsequent interpretation of the primary impact evaluation at study end. The analysis will also consider key design features for future mHealth interventions (including future iterations of an app) that may be suitable for pilot testing.

We will report on ‘reach’ of the app (proportion recruited to the trial amongst those potentially eligible for inclusion) and ‘dose’ of the app (actual logins per week as a proportion of that expected [e.g., 6 days per week for 100% adherence to intervention]).

We will describe the intervention according to the Template for Intervention Description and Replication (TIDieR) framework, as this enables reproducibility of interventions [21]. We will report our process evaluation findings according to the Tailored Implementation of Chronic Diseases checklist—and any additional emergent themes such as app design features—as this is a comprehensive framework which includes key health system factors (such as patient needs; social, cultural and political considerations; resources; and health worker interactions) [16]. Findings will be shared with relevant stakeholders from the Vietnamese National TB Program and with the broader scientific community through publications.

### Ethical considerations

Ethical approval for the process evaluation was obtained from the University of Sydney Human Research Ethics Committee (HREC) on 13 September 2023 as a modification to the overarching V‐SMART trial protocol (2019/676). Written consent (signed digitally or by hand) will be sought from participants for individual in‐depth interviews and for the process mapping exercise. Participant information sheets and consent forms will be sent to participants prior to the interview via post or weblink sent by email or SMS. eConsent will be enabled and managed through the University of Sydney's REDCap database platform that currently hosts the database for the V‐SMART Trial (Trial registration: ACTRN12620000681954).

Where it is inconvenient to the participant to attend the interview in‐person, but the participant has nominated to receive the study document by post, verbal consent will be sought if the participant has not yet been able to return their signed consent form prior to the interview. Verbal consent will be audio recorded even if the participant does not give permission for the interview to be audio recorded. The interviewing researcher will explain this to the interview participant prior to recording consent.

Participants will be assured of the confidentiality of their interview accounts, and interviews conducted in a quiet and confidential location. Pseudonyms or anonymised study identifiers will be used throughout reporting of the interviews.

## DISCUSSION

We present a detailed mixed methods process evaluation protocol of a digital health interventional trial targeting better RR/MDR‐TB adverse event management in seven provinces in Vietnam. Data collection is underway. The process evaluation is particularly important in the setting of COVID‐19 related disruptions to TB services and ongoing evidence of low uptake of the app. It will complement the economic evaluation to inform potential sustainability of future mHealth interventions within the Vietnamese National TB Program and similar settings.

A key strength of the process evaluation is data collection in parallel to ongoing trial participant follow‐up—this minimises recall bias, whilst offering an independent perspective of implementation prior to analysing the trial's primary endpoints. Another strength is representation of multiple provinces in Vietnam, each with unique patient demographics and health service considerations. Moreover, the process evaluation offers an opportunity to understand user experiences before and after app modifications. Key limitations are being unable to capture representative information from all levels of the health system (especially primary care) in all participating provinces. We are also unable to obtain perspectives of health workers and patients in non‐study sites, limiting the generalisability of our findings.

Findings will aid interpretation of the quantitative impact evaluation (composite endpoint of treatment completion or bacteriological cure at 24 months). The process evaluation will also contribute to a qualitative understanding of programmatic changes to management of RR/MDR‐TB that may have influenced incidence of adverse events and/or adverse event reporting in Vietnam: these include the rollout of WHO‐recommended, and better tolerated, all oral shorter‐course bedaquiline‐containing RR/MDR‐TB regimens (with a consequent reduction in incidence of serious adverse events than previously experienced in the National TB Program with traditional toxic regimens) whilst the trial was ongoing.

Despite the promise of mHealth technologies at improving access to care amongst those living in remote areas [22], and as a means of supporting global TB control [23, 24], due consideration must be given to the local context in which they are implemented. This is particularly the case for resource‐limited settings where there may be limited smartphone literacy (amongst patients) or a reluctance amongst health workers to embed digital technologies within mostly paper‐based clinical information systems. The V‐SMART trial process evaluation will inform feasibility of implementing such interventions with potential applicability beyond RR/MDR‐TB adverse event management (such as disease severity triaging, tuberculosis preventative therapy, or medication adherence) [25, 26, 27] within routine health services, particularly in settings where digital health infrastructure may be in its infancy. It will identify essential areas for health system preparedness for sustainability of such interventions, and important app design features that ultimately reduce health worker burden and optimise patient engagement with health care to end TB.

## AUTHOR CONTRIBUTIONS

HMY, SB, DTH and DD conceived the study and wrote the protocol with intellectual input from JN, GJF, TTHD, BHN, TTD, TNBN, TAN, and DV. HMY wrote the first draft of the manuscript and collated co‐author input. All authors revised and approved the final manuscript.

## FUNDING INFORMATION

No additional funding support was sought for the process evaluation. The V‐SMART parent trial is jointly funded by the National Health and Medical Research Council (NHMRC, APP 1157643) and the National Foundation for Science and Technology Development (NAFOSTED) (Grant number NHMRC.108.02‐2018.01). The trial sponsor is the Woolcock Institute of Medical Research, Australia.

## CONFLICT OF INTEREST STATEMENT

The authors declare they have no competing interests.
